# Chromosomal-level genome assembly of the high-quality Xian/*Indica* rice (*Oryza sativa* L.) Xiangyaxiangzhan

**DOI:** 10.1186/s12870-023-04114-0

**Published:** 2023-02-14

**Authors:** Jiayan Liang, Leilei Kong, Xiaodan Hu, Chongyun Fu, Song Bai

**Affiliations:** Rice Research Institute Guangdong Academy of Agricultural Sciences, Guangdong Key Laboratory of New Technology in Rice Breeding, Guangdong Rice Engineering Laboratory, Guangzhou, 510640 China

**Keywords:** High-quality rice, de novo assembly, Comparative genomics analysis, Long terminal repeat retrotransposons

## Abstract

The *indica* rice variety XYXZ carries elite traits including appearance and eating quality. Here, we report the de novo assembly of XYXZ using Illumine paired-end whole-genome shotgun sequencing and Nanopore sequencing. We annotated 39,722 protein-coding genes in the 395.04 Mb assembly. In comparison to other cultivars, XYXZ showed a larger gene size including the transcripts and introns, and more exons per gene. And hundreds of ultra-long genes were also detected. A total of 4362 complete LTRs were annotated, and among them, many were located next to or in protein-coding genes including several genes related to rice quality. We observed the different distributions of LTRs in these genes among XYXZ, Nipponbare, and R498, implying these LTRs might potentially affect expressions of the proximal genes and rice quality. Overall, This chromosome-length genome assembly of XYXZ provides a valuable resource for gene discovery, genetic variation and evolution, and the breeding of high-quality rice.

**Supplementary Information:**

The online version contains supplementary material available at 10.1186/s12870-023-04114-0.

## Introduction

Rice is one of the most important staple foods of the world, especially for most regions in Asia, part of North, and South America, and Africa. These regions accounted for half of the earth’s population. Benefiting from dwarf breeding and hybrid rice, the rice yield in China increased from 1.9 t/hm^2^ in 1949 to 7.0 t/hm^2^ in 2018 [1]. After achieving remarkable progress in yield, the central of rice breeding focuses more on quality. Rice quality contains four aspects: appearance, grinding, eating, and nutrient. High-quality rice is being traded at a higher price in the local and global markets [2]. The quality of rice grain mainly includes five aspects: appearance quality, milling quality, cooking quality, nutritional quality, and eating quality [3]. The specific content includes transparency, chalkiness, grain shape, amylose content, gel consistency, gelatinization temperature, protein content, and whether there is fragrance [4]. So far, many QTLs related to rice quality traits have been mapped, but few genes have been characterized, due to the genetic complexity and environmental sensitivity of these traits.

Rice has been long-standing cultivated over the planet, from tropical to temperate, and from basin bottom to high altitude mountains, the huge ecological environment difference has created the genetic diversity of rice ecotypes [5]. To require more complete knowledge of the *Oryza sativa* gene pool, 3000 rice genomes were resequenced [5, 6]. In addition, several high-quality genome assemblies of rice varieties and species had been reported, including Nipponbare, Shuhui498 (R498), IR64, Zhenshan97 (ZS97), Minghui63 (MH63), *Oryza granulate*, *Oryza longistaminata* [7–11]. Genetic and comparative analysis of these genomes serves as a foundation for the large-scale discovery of novel alleles for important rice phenotypes, using various bioinformatics and/or genetic approaches. Despite the great progress in rice breeding and research that had been made in yield and stress resistance traits, achievement in rice quality remains limited. The study of the genome structural characteristics, evolution, and expression regulation of high-grade rice will contribute to precise breeding.

Almost all eukaryotes’ genomes compose high copy numbers of transposable elements (TEs) [12]. TEs are generally able to autonomously change locations within the plant genome through both copy-paste (retrotransposon) and cut-and-paste (DNA transposon) mechanisms [13]. TEs contribute to genetic diversity among species and potentially within species in many ways. For example, TEs situated near the functional region are not under selective pressure; therefore, may have the potential to generate mutations or attain novel functions [14]. Activities of TE may cause gene breakage; genome rearrangement within a certain range, and may also affect the expression of adjacent genes, thus affecting the structure and function of the plant genome [15, 16]. A high abundance of TE copies might hinder the generation of good-quality genome assemblies, with extensive fragmentation of TE-rich regions [17].

The Xiangyaxiangzhan (XYXZ) cultivar (*Oryza sativa* ssp. *indica*) is one of the varieties of “Guangdong SIMIAOMI” and has been welcomed by farmers since it launched, with an accumulated planting area of over 200,000 ha in south China. This fragrant cultivar is famous for its long, slender grains with excellent eating quality. Tens of derivative lines and hybrid lines had been developed based on this excellent tasted cultivar. However, like other cultivars with superior grain quality, the XYXZ exhibits inferior agronomic performance, relatively low yield, and is highly prone to environmental conditions [2]. In this article, we report the de novo genome of XYXZ using Illumina and Oxford Nanopore sequencing. A high-quality reference genome was assembled, and annotation for 39,722 protein-coding genes was provided. The availability of the XYXZ genome will lay a strong foundation for both basic rice research and breeding by design.

## Result

### De novo assembly of XYXZ genome sequence

The genome of *indica* rice cultivar XYXZ was sequenced on Illumina PE150 platform and generated 158.7 million raw reads, equivalent to 47.62 Gb (~ 110× coverage) pair-end reads. We estimated the genome size of XYXZ using *k*-mer frequency distribution (*k* = 17). The estimated size of the genome was 420.7 Mb, with a 0.07% heterozygous rate and 47.16% repeat rate. Subsequently, high-quality paired-end reads were assembled into scaffold using SOAPdenovo [18] with *k*-mer =41). The XYXZ assembly had 160,915 scaffolds in total, the maximum length and the N50 of the scaffolds were 144,615 bp and 12,179 bp (Table 1).

**Table 1:** Statistics for XYXZ genome assembly

Feature	Statistics
a: Illumina	
Raw reads	158,739,841
Estimate size (Mb)	420.7
Heteozygous (%)	0.07
Repeat rate (%)	47.16
Contig number	203,121
Contig max length (bp)	87,885
Contig N50 length (bp)	8445
Total lengths of contig (bp)	310,631,206
Scaffold number	160,915
Scaffold max length (bp)	144,615
Scaffold N50 length (bp)	12,179
Total lengths of scaffold (bp)	314,991,948
b: ONT	
Mean read length (bp)	29,477
Median read length (bp)	28,331
Number of reads	1,134,686
Read length N50 (bp)	47,718
Total bases:	33,446,748,518
c: Illumina+ONT	
Scaffold Total length (bp)	395,038,024
Scaffold number	28
Scaffold max length (bp)	34,798,864
Scaffold N50 length (bp)	25,590,942

To fill the gaps in the draft genome assembly of XYXZ, we sequenced the XYXZ genome using PacBio Sequel II platform with 20× coverage and acquired 230.57 Gb subreads (Table S1). However, we tried to assemble the genome with different software or version yet fail in obtaining an ideal contig N50 (Table S2). We suspected large structural variations (SVs), or long repeat sequences could be important reasons causing smaller contig N50 in the genome assembly. Thus, Illumina short reads were mapped onto the rice genome of the *indica* cultivar Shuhui498 and the *japonica* cultivar Nipponbare [19, 20]. It resulted in a high mapping rate of 99.29 and 98.69%, respectively (Table S3), which indicated no large SVs existed in the XYXZ genome since the mapping rate should not reach 95% due to the formation of large gaps. To evaluate the assembly continuity, we introduced the LAI metric (LTR assembly index) which is independent of genome size, genomic LTR content, and gene space evaluation metrics [21]. The LAI of the draft XYXZ genome was 21.19 (LAI ≥ 20, gold quality), in other words, LTRs were well assembled. The insertion size of SMART library was about 20 kb. Is it possible that the XYXZ genome contained LTRs longer than 20 kb where SMART sequencing could not stride across?

To confirm the above hypothesis and consider the short length of contig might cause a disadvantage to the subsequent gene structure prediction and exchange rate calculation, we carried out Nanopore sequencing using the PromethION platform of Oxford Nanopore Technologies (ONT) which provided longer reads. The resulting subreads size was 33.45 Gb covering ~ 83.63 × of the genome. The mean and the N50 of the reads’ length were 29,477 bp and 47,718 bp. The reads were assembled with nextdenovo (https://github.com/Nextomics) and corrected with Racon [22] producing error corrected contigs amounting to 1,134,686. The assembled contigs were merged with the Illumina clean reads for further error correction using Pilon [23]. This process yielded assembly results with a contig/scaffold N50 length of 25.59 Mb and a total length of 395.04 Mb (Table 1).

The completeness and accuracy of this genome were assessed with three approaches. First, we used the software tool BUSCO [24], which indicated that 1596 (98.9%) of the 1614 highly conserved core proteins in the embryophyta lineage were revealed. Second, CEGMA [25] showed 245 (98.79%) of 248 core eukaryotic genes were present in our XYXZ genome. Third, we mapped all the high-quality reads from short-insert-size libraries back to the assembly using BWA (Burrows-Wheeler Aligner) [26], it showed good alignments with an average mapping rate of 99.6% and coverage of 99.56% (Table S4). Taken together, these results indicated that the genome assembly is of high quality.

**Fig. 1:**
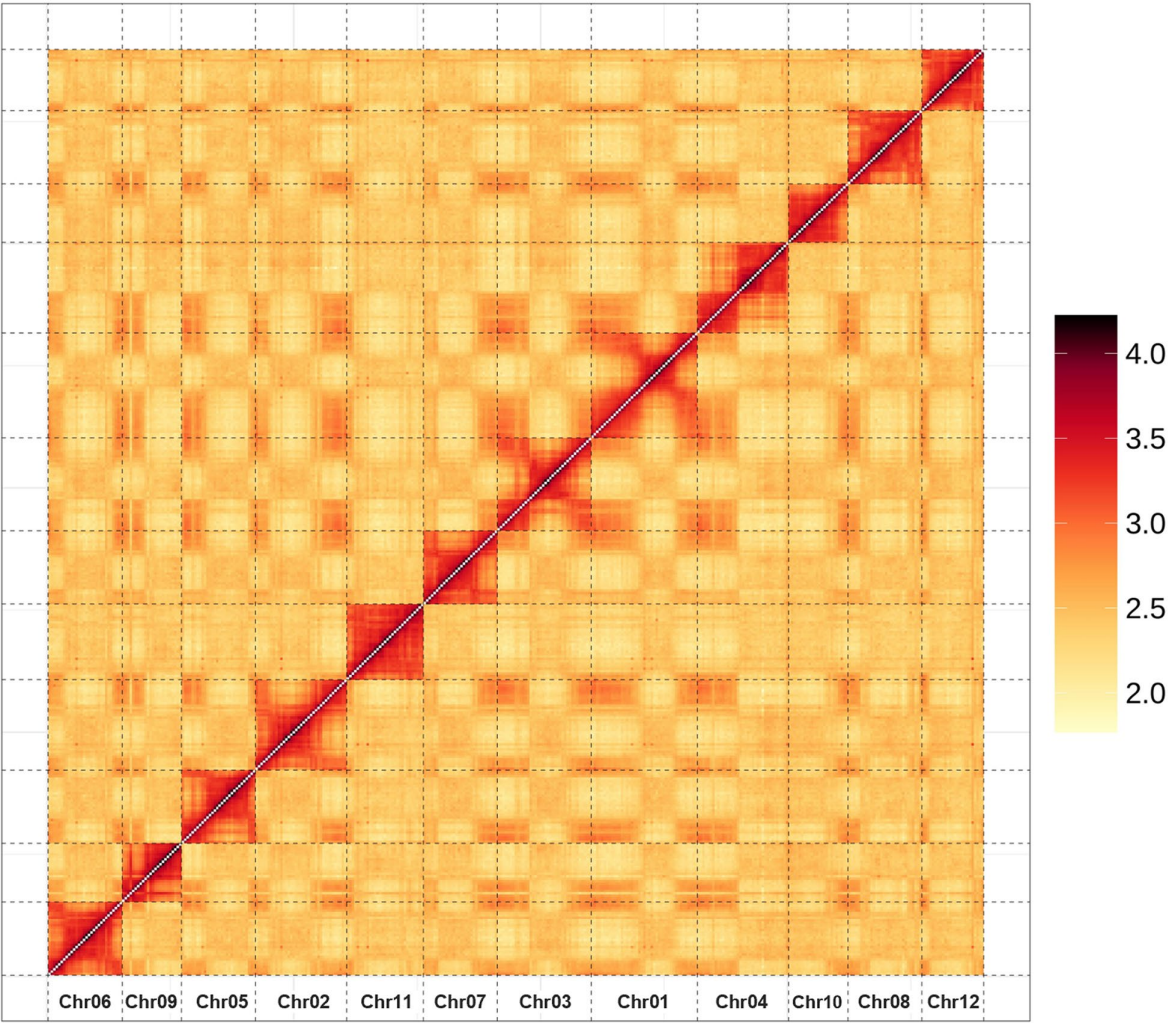
Hi-C scaffolding of the XYXZ genome. Hi-C interaction matrices show the pairwise correlations between ordered scaffolds along the 12 pseudomolecules. The intensity of the dark color is proportional to the strength of the correlation

Hi-C (High-throughput chromosome conformation capture) was employed to assist in assembling the contigs/scaffolds to chromosome scale. Finally, the scaffolds were constructed into 12 pseudochromosomes with a 99.21% mounting rate, with only 3.11 Mb unplaced sequences. The sequence length of the pseudochromosomes ranges from 25,015,458 bp to 44,279,745 bp (Fig. 1, Table 2).

**Table 2:** Basic sequence statistics of XYXZ

Chromosome	Sequence ID	Cluster Number	Sequence Length (bp)
Chr01	Hic_asm_7	3	44,279,745
Chr02	Hic_asm_3	2	37,804,255
Chr03	Hic_asm_6	2	38,898,513
Chr04	Hic_asm_8	9	37,484,607
Chr05	Hic_asm_2	2	30,874,169
Chr06	Hic_asm_0	3	31,804,368
Chr07	Hic_asm_5	2	30,582,228
Chr08	Hic_asm_10	2	30,330,803
Chr09	Hic_asm_1	2	25,015,458
Chr10	Hic_asm_9	3	25,568,435
Chr11	Hic_asm_4	2	32,490,898
Chr12	Hic_asm_11	2	26,792,270

### Gene annotation and repeat analysis

We employed a combined strategy to identify repeats in the genome based on homology alignment and de novo search. Tandem Repeat and repeat regions were extracted using TRF (http://tandem.bu.edu/trf/trf.html) and RepeatMasker software (http://www.repeatmasker.org), respectively. Raw TE library was predicted with LTR_FINDER [27], RepeatScout, and RepeatModeler. The annotation showed that approximately 44.89% (177.33 Mb) of non-redundant regions of the assembled genome consisted of repeat sequences (Table S5).

Three methods were employed to predict protein-coding genes of the XYXZ genome, including ab initio prediction, homology-based, and RNA-seq-aided gene predictions. For ab initio gene prediction, Augustus (http://augustus.gobics.de/), GlimmerHMM (http://ccb.jhu.edu/software/glimmerhmm), SNAP [28], Geneid [29], Genscan [30] software were adopted, and each predictor was trained with the RNA-seq dataset acquired from 4 tissues (root, stem, leaf, panicle) in advance. Five well-annotated rice varieties including three *Oryza sativa* ssp. *indica* (MH63, R498, ZS97), Nipponbare (*Oryza sativa* ssp. *japonica)*, and *O. rufipogon* were taken as homologous to conduct homology-based gene prediction. All genes’ structures obtained from the above methods were combined using EVidenceModeler (http://EVidenceModeler.github.io). In total, 39,722 protein-coding genes were predicted in the XYXZ genome. Interestingly, the average transcript length of XYXZ is 2986 bp, which is longer than other cultivated rice while shorter than *Oryza rufipogon*, and so is the average intron length. The average exons per gene in XYXZ is also larger (Fig. S1, Table S6). Approximately 96.08% (38,164) of these gene models were supported by RNA-seq and/or homologous proteins (Fig. S2).

Next, we aligned the predicted protein sequences to Swiss-Prot protein databases, Non-Redundant (NR), the Integrated Resource of Protein Domains and Functional Sites (InterPro), and the Kyoto Encyclopedia of Genes and Genomes (KEGG) database for gene functions inference. We also identified motifs and domains within the genes using PFAM database. In total, 92.30% of predicted protein-coding genes could be functionally annotated (Tabel S7).

Non-coding RNA genes including transfer RNA (tRNA) genes, ribosomal RNA (rRNA) genes, small nuclear RNA (snRNA) genes, and microRNA (miRNA) genes were known to be involved in important biological processes. We used tRNAscan-SE program (http://lowelab.ucsc.edu/tRNAscan-SE/) to predict tRNAs. And the highly conserved rRNAs were predicted using Blast with relative species’ rRNA sequences as references. For miRNAs and snRNAs, we employed the infernal software (http://infernal.janelia.org/) to search against the Rfam database with default parameters. A total of 6728 miRNA genes, 638 tRNA genes, 1468 rRNA genes, and 1374 snRNA genes in the XYXZ genome were annotated (Table S8).

### Genomic variations between XYXZ and other rice varieties

**Fig. 2:**
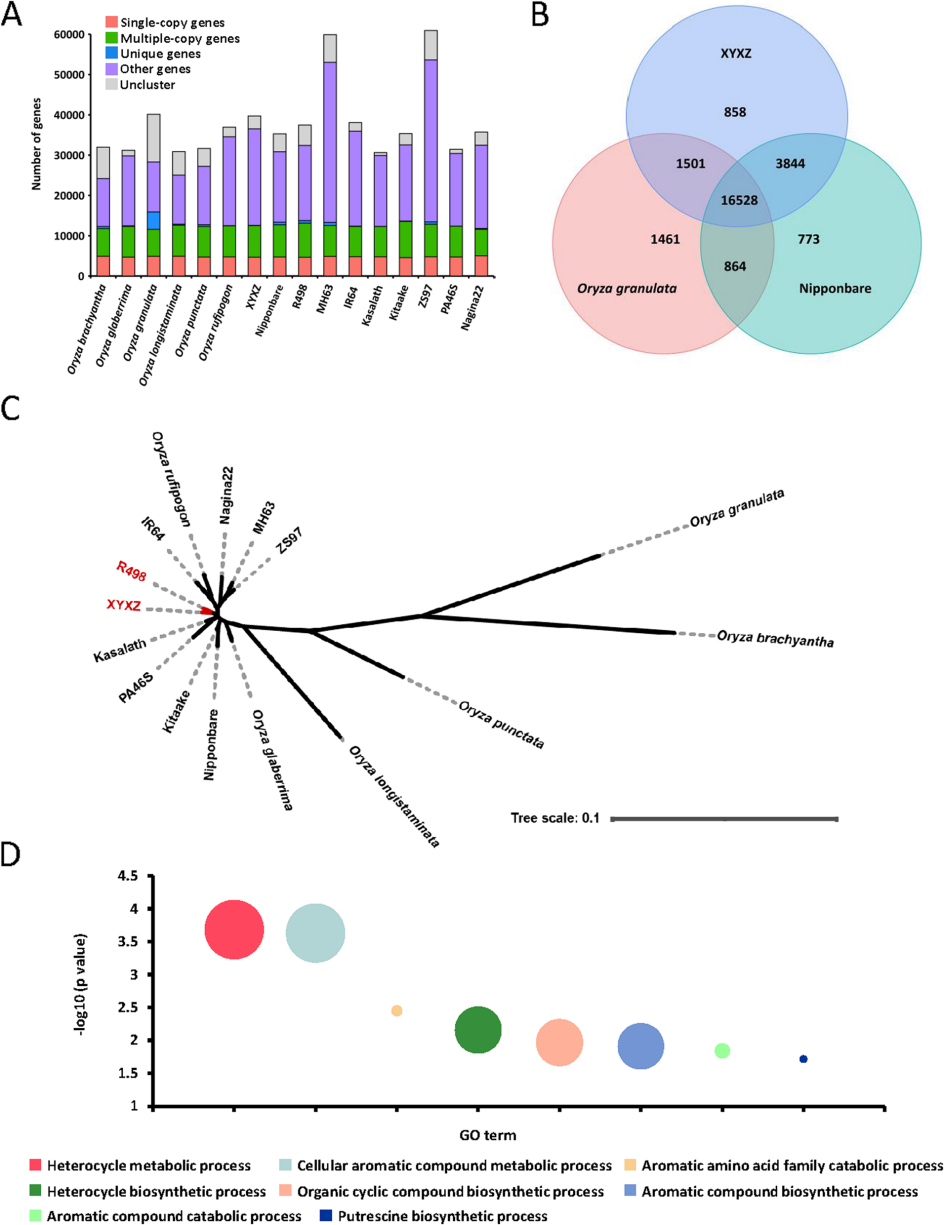
Comparative genomics analysis between XYXZ and other *Oryza* species. **A** Gene family comparison between XYXZ and other *Oryza* species. **B** Venn diagram showing common and unique gene families between XYXZ, Nipponbare, and *O. granulata*. **C** Phylogenetic tree for XYXZ. Phylogenetic tree constructed with 3740 single-copy gene families in the maximum likelihood method. **D** Scatterplots showing the enriched aroma-related GO biological process of positively selected genes in XYXZ

Based on predicted protein-coding genes, we identified 22,731 gene families in XYXZ. Through the clustering analysis with 15 other *Oryza* genomes, a total of 53,515 gene families were clustered. Among them, 6985 gene families were found in all *Oryza* genomes. Single-copy genes count 63.32% (25,151 genes) of the protein-coding genes in XYXZ (Fig. 2A). Over 70% of gene families (16,528) were shared by *Oryza granulate* and Nipponbare (Fig. 2B). We identified 858 gene families (2505 genes) that are only present in XYXZ but are absent in the other two genomes. Analysis of GO terms for these specific families revealed that several functional pathways involved in negative regulation of translation (GO:0017148), Box C/D RNP complex (GO:0031428), and RNA glycosylase activity (GO:0030597) are enriched in XYXZ (Table S9). Phylogenetic analyses showed that XYXZ had a close relationship with R498 (Fig. 2C).

The expansion and contraction gene families of XYXZ were estimated using CAFE. A total of 2940 genes expanded in XYXZ, while the contracted genes were 642 (Tables S10 and S11). Significantly expanded families (*p* ≤ 0.05) were clustered in the GO items. Among the expanded genes, the most enriched molecular function was carbohydrate binding (GO:0030246) (Table S12). As for contracted genes, the most enriched molecular function was catalytic activity (GO:0003824) (Table S13). These genes may be involved in specific phenotypes and environmental adaptability.

In addition, we used MUSCLE software (http://www.drive5.com/muscle/) and branch-site model in PAML software to detect signals of positive selection on genes involved in adaptive divergence and/or human selection in XYXZ, and the five closely related rice genomes of R498, Nagina22, ZS97, MH63, and IR64 were served as background branches. As a result, 703 positive selective genes were identified (Table S14). Aroma is one of the excellent qualities of XYXZ, over 300 compounds have been recognized from aromatic rice including lipids, aldehydes, heterocycles, alcohols, and phenols [2]. Sixty-six of the positive selective genes were annotated to be involved in the heterocycle metabolic process, such as lipoyl synthase, methyltransferase, and pyridoxal kinase. It suggests that these genes may have undergone artificial selection during the breeding (Fig. 2D).

### Transposable elements in XYXZ

**Fig. 3:**
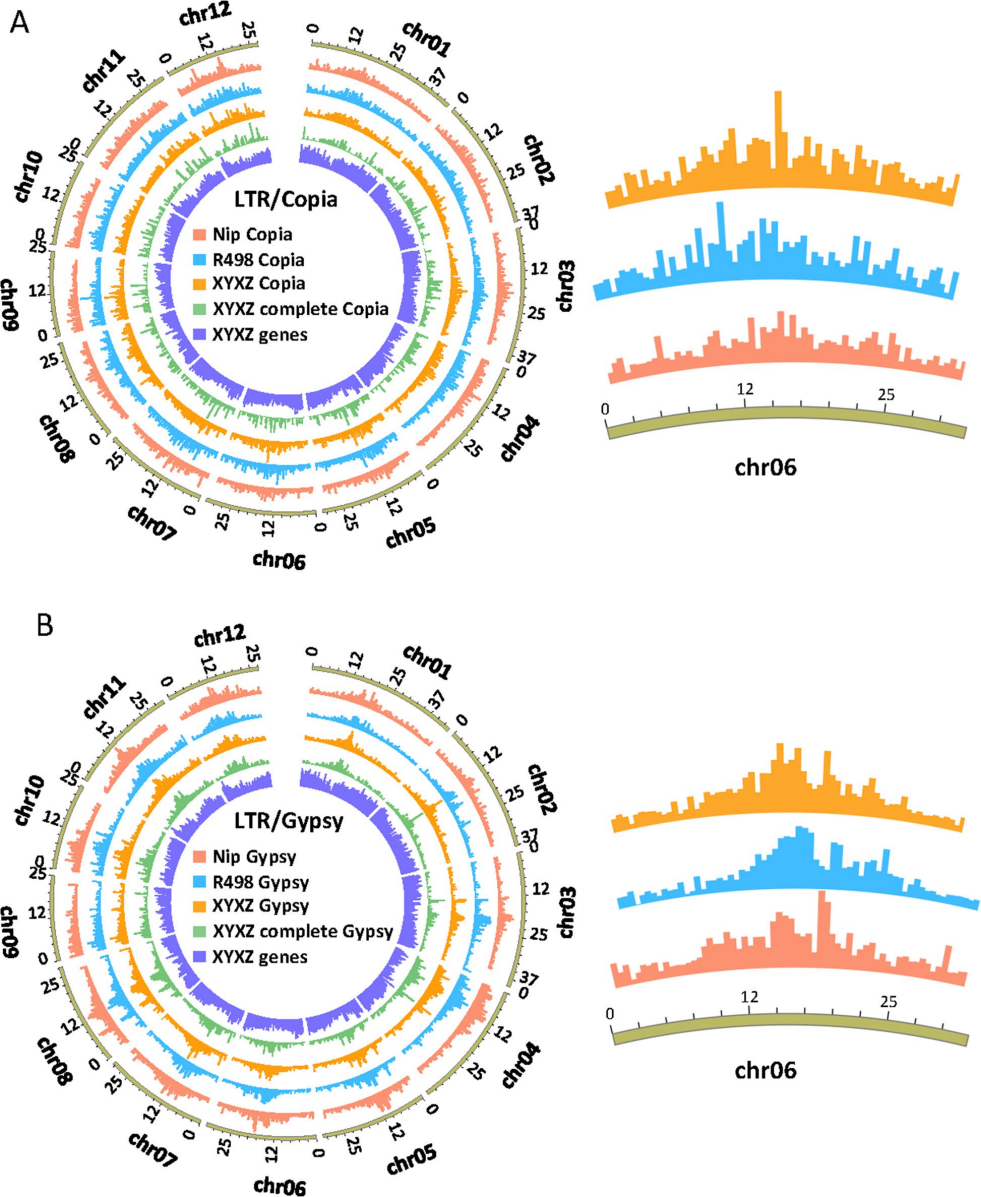
Distribution of LTRs (Copia and Gypsy) along the chromosomes of the XYXZ, R498, and Nipponbare. Tracks from the outer to inner circles indicate the following: 1, Chromosome; 2, All predicted LTRs in Nipponbare; 3, All predicted LTRs in R498; 4, All predicted LTRs in XYXZ; 5, LTRs with complete structure in XYXZ; 6, Predicted genes in XYXZ. **A** Copia. **B** Gypsy

TEs are widely spread in the plant genome and provide a rich source of new genes and a regulatory sequence [16]. Given the important role of TEs, we extend the analysis of TEs in XYXZ and made a comparison to that of its close relative *Oryza*. The six genomes contain a similar amount of transposable elements ranging from 51.63 to 56.94% of the total genome length (54.34% for XYXZ) (Table 3, Table S15). These TEs are classified into 12 superfamilies according to the hierarchical TE classification system [28]. In the XYXZ genome, approximately 70% of the TEs are classified into class I elements (retrotransposon). The percentages of each TEs superfamilies between XYXZ and R498 are close. For example, XYXZ had 22.67 and 4.75% LTR/Gypsy and LTR/Copia, respectively, while R498 had 21.72 and 4.77%, respectively (Table 3). Despite the similar content of LTRs in the genomes, their distribution is varied. Figure 3 shows the distribution of Copia and Gypsy LTRs in the XYXZ, R498, and Nipponbare genomes.

**Table 3:** The characteristics of TEs in the genomes of XYXZ, R498, and Nipponbare

	XYXZ			R498			Nipponbare		
	Length (bp)	Percentage of genome (%)	Number	Length (bp)	Percentage of genome (%)	Number	Length (bp)	Percentage of genome (%)	Number
**Class I: Retrotransposon**	152,593,462	38.63%	293,791	150,737,098	38.55%	292,231	135,624,823	36.34%	309,239
**LTR-Retrotransposon**	142,183,026	35.99%	252,194	140,192,746	35.86%	250,822	124,237,591	33.29%	265,728
LTR/Gypsy	89,538,544	22.67%	106,882	84,915,354	21.72%	106,875	71,753,076	19.22%	109,588
LTR/Copia	18,783,908	4.75%	38,386	18,653,262	4.77%	37,029	20,886,614	5.60%	46,509
LTR/Other	33,860,574	8.57%	106,926	36,624,130	9.37%	106,918	31,597,901	8.47%	109,631
**Non-LTR Retrotransposon**	10,410,436	2.64%	41,597	10,544,352	2.70%	41,409	11,387,232	3.05%	43,511
SINE	880,763	0.22%	4482	1,005,769	0.26%	4644	929,204	0.25%	4559
LINE	9,529,673	2.41%	37,115	9,538,583	2.44%	36,765	10,458,028	2.80%	38,952
**Class II: DNA Transposon**	71,167,668	18.02%	287,904	70,466,702	18.02%	284,836	69,813,358	18.70%	277,817
EnSpm/CACTA	2,860,768	0.72%	5146	2,624,568	0.67%	5080	3,760,181	1.01%	5443
hAT	5,738,901	1.45%	22,713	4,539,718	1.16%	16,976	6,377,976	1.71%	25,049
Harbinger	250,601	0.06%	957	268,432	0.07%	1051	247,626	0.07%	933
Tc1/Mariner	10,260,759	2.60%	51,207	9,369,588	2.40%	49,076	8,890,174	2.38%	46,603
MuDR	186,268	0.05%	809	173,445	0.04%	778	167,849	0.04%	749
Helitron	3,320,855	0.84%	9233	3,410,449	0.87%	9754	3,571,714	0.96%	7947
Other	48,549,516	12.29%	197,839	50,080,502	12.81%	202,121	46,797,838	12.54%	191,093
**Unclassified**	30,320	0.01%	265	27,697	0.01%	241	29,044	0.01%	253
**Total content**	214,682,827	54.34%	588,740	209,087,702	53.48%	595,353	192,700,449	51.63%	584,921
**Total Genome Length**	395,040,224			390,983,850			373,245,519

TEs are closely related to the variation of genetic traits, the insertions of TEs contain the potential for both genetic and epigenetic influences on the flanking genes [29]. The insertion of TEs into coding regions may cause direct influences on gene function, in addition, they also may insert into existing regulatory regions or create new regulatory elements, resulting in altered gene expression [30]. A typical LTR retrotransposon contains specific signals for packaging, dimerization, reverse transcription, and integration [28]. It can be inferred that the expression of those flanking genes might be affected by the LTRs. Since the identification of LTRs was based on the presence of characteristic LTRs, we filter the structural uncomplete LTRs to further investigate LTRs in XYXZ. Out of these sets, 4362 complete LTRs were found, consisting of 2818 LTR/Gypsy, 643 LTR/Copia, and 901 LTR/Other (Fig. S3). Six hundred and seventy-four of these LTRs are located within 1 kb upstream of 569 genes while 655 LTRs position downstream of 646 genes (Table S16). Thirty-one of these flanking genes had been functionally studied, mainly relating to plant morphogenesis, stress response, nutrient utilization, and rice quality (Table 4). Half of the plant morphogenesis genes participate in internode length and lignin biosynthesis, expression of these genes might be regulated by the flanking LTRs and thereby affect the stem strength of XYXZ. The result shows that 8 stress/resistance genes are involved in biotic stress (including blast resistance, leaf blight resistance, and stripe disease resistance) and abiotic stress (including heat resistance, drought tolerance, and salt stress response). In terms of nutrient utilization, three genes related to the iron content and phosphate content locate downstream and two nitrogen-utilize genes are situated upstream of complete LTRs.

**Table 4:** Some cloned genes containing complete LTR in their 1 kb flanking regions

Category	Relative position of LTR	Symbol	Gene function or phenotypic characteristics
A^a^	Upstream	*D35*	Internode length
	Upstream	*THIS1*	Lipase; tillering, plant height, and spikelet fertility
	Upstream	*ACL2*	Abaxially curled leaf 2
	Upstream	*Os4CL4*	Lignin biosynthesis, plant cell wall modification
	Downstream	*NAL1*	Number of adventitious roots, internode length, chlorophyll content, leaf shape, leaf width, grain yield, spike type, Plant cell size
	Downstream	*D10*	Establishment of internode development model, biosynthesis of monocytolide, regulation of tiller formation
	Downstream	*TIG1*	Tiller angle, plant cell length
	Downstream	*CYP714B1*	Internode length
	Upstream	*OsMADS4*	Gene expression regulation, ear morphogenesis, stamen development
A/B^b^	Upstream	*DDX47*	Number of lateral roots, plant height, root length, heat resistance
	Upstream	*D1*	internode length, leaf width, leaf length, grain size, and red-light sensitivity Gibberellin sensitivity, drought tolerance, stem diameter, 1000 grain weight, spike density
	Downstream	*OsVAMP714*	Rice blast resistance, plant height, shoot growth rate, leaf sheath length
	Downstream	*HTD12*	Internode length, tiller number, cold tolerance, drought tolerance, abscisic acid content, β- Carotene content, Osmotic regulation capacity, salt resistance
B^c^	Upstream	*OsDi19–4*	Drought-induced 19 gene
	Upstream	*OsGDI3*	Response to fungi, anti-bacterial response, low temperature stress response, water stress response, insect resistance response, salt stress response
	Downstream	*DEPG1*	Bacterial leaf blight resistance, rice bacterial stripe disease resistance
	Downstream	*OsCK1*	Heading stage, osmotic stress sensitivity, abscisic acid sensitivity, salt tolerance
C^d^	Upstream	*OsYSL15*	Iron (III)-deoxymugineic acid transporter
	Upstream	*OsPht1;1*	Phosphate transporter gene, phosphorus absorption, phosphate content
	Upstream	*OsSPX4*	Phosphate content
	Downstream	*OsAAP3*	Tiller number, spike number, amino acid content, grain number per plant, yield per plant, nitrogen utilization efficiency
	Downstream	*OsNRT1.1A*	Plant height, heading date, aboveground biomass, nitrate transport, yield per plant, nitrogen utilization rate
D^e^	Upstream	*AL1*	Aleurone layer and/or transfer cell-specific gene
	Upstream	*OsGS1;3*	Interact with Nf-yc12 to regulat the synthesis of protein in the endosperm
	Upstream	*OsPDIL1;1*	Endosperm color, silty endosperm
	Downstream	*OsPK2*	Protein content, seed development characteristics, amylose content, starch content, total lipid content, chalky endosperm, Germination rate, 1000 grain weight
	Downstream	*GGC2*	Spike length, grain length, 1000 grain weight
E^f^	Upstream	*OsUGT707A2*	UVB stress response, flavonoid biosynthesis
	Downstream	*RFT1*	Heading stage, photoperiod sensitivity, growth stage
	Downstream	*OsNDPK2*	Leaf color, chlorophyll content, chloroplast development
	Downstream	*RSS3*	Cell length, root

^a^Plant morphogenesis

^b^Plant morphogenesis/Stress response

^c^Stress response

^d^Nutrient utilization

^e^Rice quality

^f^Other

**Fig. 4:**
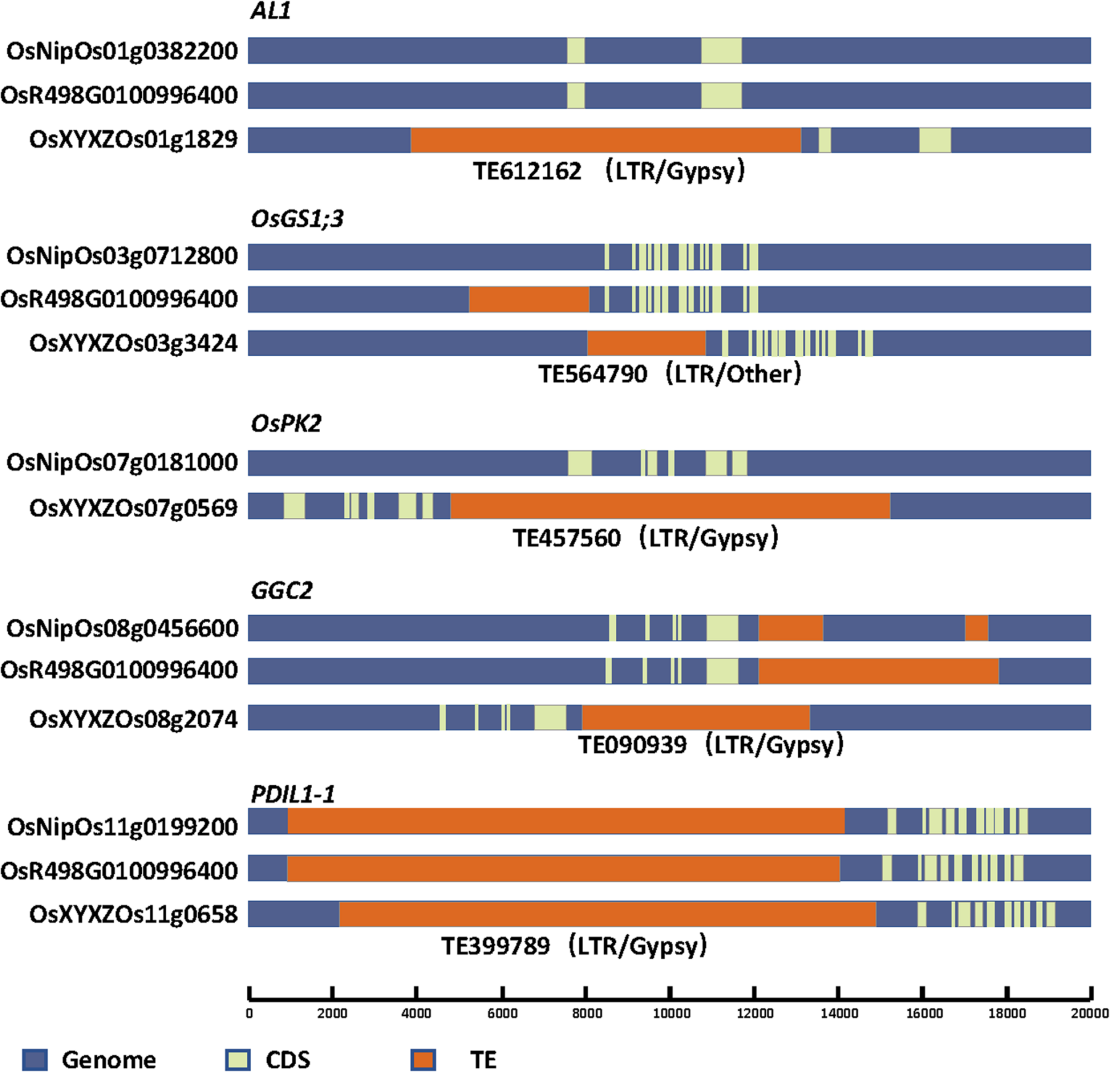
The structure characteristics of several quality-related genes in genomes of XYXZ, R498, and Nipponbare. *AL1* (*Aleurone layer and/or transfer cell-specific gene*); *OsGS1;3* (*Glutamine synthetase*); *OsPK2* (*Plastidic pyruvate kinase*); *GGC2* (*G protein gamma subunit*); *PDIL1–1* (*Protein disulphide isomerase-like enzyme*)

Five of the LTR flanking genes are related to rice quality regulating grain shape, filling process, and/or endosperm substance. To visually display the location relationship between LTRs and these genes, we compared their structure models among XYXZ, Nipponbare, and R498 (Fig. 4). *AL1* is an aleurone layer and/or transfer cell-specific gene, only expressed in the aleurone layer of immature seeds [31]. According to our analysis, a complete LTR/Gypsy is located within 1 kb upstream of *AL1* in XYXZ which is absent in the other two varieties. *OsGS1;3* is one of the glutamine synthetases in rice, *NF-YC12* is directly bound to its promoters to regulate endosperm development and the accumulation of storage substances in rice seeds [32, 33]. An LTR/Other inserts in the upstream of *OsGS1;3* in XYXZ and R498, no such insertion was found in Nipponbare. *OsPK2* regulates rice endosperm starch synthesis, compound granule formation, and grain filling [34]. An LTR/Gypsy was found downstream of *OsPK2* in XYXZ which does not produce a good alignment in Nipponbare, no corresponding gene of *OsPK2* is predicted in R498. *GGC2* determines grain size in rice [35], the structure model in the two *indica* rice are identical, however, the sequence of TE090939 (LTR/Gypsy) is not completely matched in Nipponbare genome region. *PDIL1–1* controls endosperm development through regulation of the amount and composition of seed proteins in rice [36]. The three varieties contain the same LTR/Gypsy upstream of *PDIL1–1*.

## Discussion

As a genomic model crop species for monocots and the main staple food for humans, rice genetics has aroused great interest [37]. It has become clear that one single genome is not enough to represent the huge amount of variation in rice genomes [38]. The previously published de novo assemblies, for example, Nipponbare and 93–11, had facilitated the scientific research and breeding of rice in aspects of yield, disease resistance, and stress resistance, while inadequate in rice quality.

Here we report the de novo assembly and annotation of XYXZ, a south China cultivar rice with excellent appearance and eating quality. The total length of the assembly is 395.04 Mb, we predicted that the XYXZ genome contains 39,722 protein-coding genes (Table S6). The availability of a high-quality genome and annotation for XYXZ will be useful for associating traits of interest with genetic variations, and for identifying the genes controlling those traits, especially in rice quality.

XYXZ also contains ultra-long LTRs in its genome. SMART and Nanopore are third-generation sequencing technology and are increasingly used in many different research areas [39]. They rely on different principles, assembly with either PacBio or Nanopore reads, followed by polishing with Illumina reads, which facilitated high-quality genome reconstruction [40]. We chose PacBio at first for it had been successfully used in several *Oryza* genomes [7, 9, 20, 41, 42]. We assembled several version draft genomes using Illumina and PacBio reads, but all of them were far from satisfactory for the small size in contig N50 (Table S2). However, no large SVs existed in the XYXZ genome (Table S3), and LAI indicated that LTRs were well assembled. Thus, we proposed the XYXZ genome may contain many ultra-long LTRs that PacBio read length could not stride over. Subsequently, we employed Nanopore sequencing to confirm the hypothesis and acquired assembly with contig N50 up to 25.59 Mb (Table 1). We found 4362 complete LTRs in the XYXZ genome (Fig. S3), 361 of which were longer than 19.2 kb, some of them even reached 38 kb (Fig. S4). The genetic and biological function of these ultra-long LTRs remains to be resolved. In comparison with other rice cultivars, the XYXZ genome does have special features. Although the CDS length in the genome of XYXZ is similar to that in other rice reference genomes, the protein-coding genes in XYXZ have larger-sized transcripts and introns, and more exons per gene (Fig. S1, Table S6). Compared with other rice reference genomes, we detected more or longer LTR types of TEs in some genes of the XYXZ genome (Fig. 4). In addition, we screened the genome and found 5466 ultra-long genes (≥5918 bp, double the average transcript length). Among these genes, 540 are inserted by complete LTRs (Fig. S5). The insertion of TEs may result in large transcripts and introns in genome evolution [43, 44], which may be one of the factors for special gene features in XYXZ.

We identify 1215 genes closely flanked (within 1 kb) by complete LTRs in their regulatory regions (Table S16), expression of these genes is probably under control by the TEs. Evidence had been shown that LTRs may provide regulatory activity [43–46]. The color of the black-skin grape is determined by the accumulation of anthocyanins, an LTR insertion upstream of *VvmybA1* blocks the expression of the gene and gives rise to the white-skin grape [43]. In blood orange, an LTR/Copia insertion at 5’UTR of *Ruby* provides regulatory sequences for initiating the expression of the *Ruby* and promoting cold-dependent expression of the gene, thereby, facilitating anthocyanin production in the fruits [44]. In XYXZ genome, five genes related to rice quality are closely adjacent to LTRs (Fig. 4). We surmise these LTRs could have a potential regulatory effect on rice quality genes, thereby affecting the appearance quality and cooking quality of XYXZ. It is known that the genetic network that determines quality traits in rice is complex with low heritability and is generally vulnerable to environmental impacts. The expressions of TE families are highly dynamic across both tissues and genotypes. Regulatory elements within TEs have been shown to influence the expression of nearby genes, either through acting as enhancers or creating merged transcripts initiated within the TE [46]. Besides, the epigenetic trade-off between TE and the proximal gene could also influence the expression of both [47]. The existence of TEs might provide hints for why quality traits suffer from large environmental effects. More studies need to be conducted in the future to elucidate the details of the regulatory relationship. It is noteworthy that the structure model of these genes in the three cultivars is not completely consistent, which not only enriches the genetic diversity of the genes but simultaneously, makes the regulatory mechanism even more complicated.

## Conclusions

The good quality genome sequence produced in the study will facilitate the biological research at the genomic level, especially for the deep mining of genetic variations in rice quality traits, and provide more information for genome-based molecular breeding in rice.

## Methods

### Plant materials and DNA/RNA extraction

Collection of plant material and all methods were performed in accordance with the relevant guidelines/regulations/legislation. The cultivar XiangYaXiangZhan (*Oryza sativa* ssp. *indica*) was grown in the greenhouse of the Rice Research Institute, Guangdong Academy of Agricultural Sciences, Guangzhou, China. Healthy and fresh tissues were harvested from the best-grown individual plant 2 months after being transplanted. For WGS sequencing, high-quality genomic DNA was extracted from leaves with a Plant DNeasy kit (Qiagen, USA). The extracted DNA was then electrophoresis on a 0.8% agarose gel and detected using a Qubit fluorometer (Thermo Fisher Scientific, USA) for examination of quality and quantity. Total RNA from root, stem, leaves, and panicle were isolated using Invitrogen TRIzol (Thermo Fisher Scientific, USA).

### Short-insert library preparation and sequencing

Genomic DNA was randomly interrupted into about 350 bp fragments using S220 Focused-ultrasonicator (Covaris, USA). Then the PE library was prepared with NEB Next® Ultra DNA Library Prep Kit (NEB, USA) following the manufacturer’s instructions. Total RNA isolated from different tissues were used to prepare strand-specific RNA seq libraries with TruSeq Stranded Total RNA Library Prep Kit (Illumina, USA). Libraries were sequenced by the Illumina NovaSeq PE150 platforms (Illumina, USA).

The raw data were filtered by removing adapter and low-quality reads. The short reads were assembled into contigs and then scaffold using Soapdenovo [18] and *k*-mer. Genome size was estimated from the *k*-mer using JellyFish-2.2.10 with a *k*-mer size of 17 (contig scale) and 41 (scaffold scale), respectively.

### PacBio sequencing and genome assembling

DNA of XYXZ was sheared to ~ 20 kb targeted size, followed by damage repair and end repair, blunt-end adaptor ligation, and size selection. Finally, the libraries are sequenced on the PacBio sequel instrument. Around 12 million subreads (Tabel S1) were used for assembly with FALCON v1, FALCON v2, Canu, hicanu, hifiasm v1, and hifiasm v2 to generate the contig.

### Nanopore sequencing and genome assembling

High-quality genome DNA was sheared using Covaris® g-TUBE™ device (Covaris, USA), and followed by repairing DNA damage. The DNA library for MinION sequencing was prepared with Rapid Sequencing Kit (Oxford Nanopore Technologies, UK). Then, the sequencing was performed on the PromethION platform (Oxford Nanopore Technologies, UK). Packages including Canu v2.0, wtdbg v2.5, Smartdenovo v1.0, Nextdenovo v2.2 (https://github.com/Nextomics), and Racon v1.3.1 [22] were employed to conduct the genome assembling. Short reads obtained from PE150 platforms were used to correct the assembled contigs with pilon v1.22 [23]. The completeness and accuracy of the genome were assessed with BUSCO (Benchmarking Universal Single-Copy Orthologs) [24], CEGMA (Core Eukaryotic Genes Mapping Approach) [25], and BWA (Burrows-Wheeler Aligner) [26].

### Hi-C sequencing and chromosomal architecture

At first, the Leaf sample was ground with liquid nitrogen and cross-linked with a 4% formaldehyde solution for 30 min. Then, 2.5 M glycine was added to stop the cross-linking reaction and neutralize the remaining formaldehyde. The nuclei were digested with the *Dpn*II restriction enzyme. The sticky ends of the digested fragments were labeled with biotin − 14-dCTP and re-ligated by T4 DNA polymerase. Next, the DNA fragments were sheared by ultrasonicator to a size of 200-600 bp. The biotin-labeled DNA fragments were enriched and then amplified by PCR to produce the Hi-C sequencing library. The Hi-C library was sequenced on the Illumina NovaSeq PE150 platform. The chromosomal-scale genome was assembled using ALLHiC [48] based on Hi-C data.

### Genome annotation

As modified based on the method reported by Du et al. [20], tandem repeat was extracted using TRF v4.09 (http://tandem.bu.edu/trf/trf.html) by ab initio prediction. The homolog prediction commonly used Repbase [49] database employing RepeatMasker v4.1.0 (http://www.repeatmasker.org) software and its in-house scripts (RepeatProteinMask) with default parameters to extract repeat regions. LTR_FINDER [27], RepeatScout v1.0.5 [50], RepeatModeler v2.0.1 were employed to build de novo repetitive elements database with default parameters. Repeat sequences (with lengths > 100 bp and gap ‘N’ less than 5%) in the database constituted a raw TE library. The de novo TE library together with Repbase was supplied to uclust to produce a non-redundant library. DNA-level repeat identification was carried out by RepeatMasker.

The gene structure de novo annotation was made using Augustus v3.2.3 (http://augustus.gobics.de/), Genscan v1.0 [30], Glimmer v3.0.4 (http://ccb.jhu.edu/software/glimmerhmm), Snap v2013.11.29 [28], and Geneid v1.4 [29]. Homology-based method was also employed to annotate the protein-coding gene structure. We built a non-redundant protein database of *O. indica* (MH63, R498, ZS97), Nipponbare (*O. japonica*), and *O. rufipogon*. Then the protein sequences were aligned to the genome by Blastall v2.2.26 (E-value cutoff by 1E-5). The blast hits were conjoined by solar. For each blast hit, Genewise was used to predict the exact gene structure in the corresponding genomic regions. RNA-seq data were mapped to the genome using Tophat v 2.0.13 (https://tophat.com). Then, cufflinks v 2.1.1 (http://cufflinks.cbcb.umd.edu/) was used to assemble transcripts to gene models. EVidenceModeler (http://EVidenceModeler.github.io) was used to combine all genes’ structures.

The tRNAs were predicted using the program tRNAscan-SE v1.4 (http://lowelab.ucsc.edu/tRNAscan-SE). Blast v2.2.26 was used to predict rRNA sequences. Other ncRNAs, including miRNAs, snRNAs were identified by searching against the Rfam v14.1 database with default parameters using the infernal software (http://infernal.janelia.org/).

### Gene family evolution analysis

OthoMCL was used to identify gene families in *O.brachyantha*, *O. glaberrima*, *O. granulate*, *O. longistaminata*, *O. punctata*, *O. rufipogon*, Nipponbare, R498, MH63, IR64, kasalath, kitaake, ZS97, PA46S, Nagina22. For multiple-transcript genes, only the transcript with the longest coding region was reserved. And filter out the genes encoding protein less than 50 amino acids or those with stop codons inside. The similar relationship between protein sequences of all *Oryza* was obtained through all vs all blast p, with default E value. The inflation of OthoMCL was 1.5.

A total of 3740 single-copy gene families are subject to MUSCLE (http://www.drive5.com/muscle/) for multi-sequence alignment, and then combine all the alignment results to form a super alignment matrix. The sequence alignment result was used to construct phylogenetic tree by RAxML (http://sco.hits.org/exelixis/web/software/raxml/index.html) with the maximum likelihood method.

Filter gene families with abnormal gene numbers in individual *Oryza*, the rest gene families were imported into CAFE software (http://sourceforge.net/projects/cafehahnlab) to analyze the amplification and contraction of gene families (family-wide *P*-value< 0.05 and Viterbi *P*-values< 0.05). MUSCLE was used to analyze positive selective genes in XYXZ with default parameters.

## Supplementary Information

**Additional file 1: Table S1.** Statistics for SMART Sequencing.

**Additional file 2: Table S2.** Statistics for draft genome assembled using Pacbio data.

**Additional file 3: Table S3.** Mapping rate of XYXZ Illumina short reads to R498 and Nipponbare.

**Additional file 4: Table S4.** Evaluation of assembly results.

**Additional file 5: Table S5.** Statistics for repeat sequence.

**Additional file 6: Figure S1.** Distribution of gene structure length.

**Additional file 7: Table S6.** Statistics for gene structure annotation.

**Additional file 8: Figure S2.** Venn diagram showing gene function annotation.

**Additional file 9: Table S8.** Statistics for non-coding RNA.

**Additional file 10: Table S9.** GO terms for specific families in XYXZ.

**Additional file 11: Table S10.** Expenssion genes in XYXZ.

**Additional file 12: Table S11.** Contraction genes in XYXZ.

**Additional file 13: Table S12.** Enriched GO terms for expansion genes in XYXZ.

**Additional file 14: Table S13.** Enriched GO terms for contraction genes in XYXZ.

**Additional file 15: Table S14.** Positively selected genes in XYXZ.

**Additional file 16: Table S15.** Statistics for TEs in 9311, MH63, and ZS97 genomes.

**Additional file 17: Figure S3.** Complete LTRs in XYXZ genome.

**Additional file 18: Table S16.** Complete LTRs and the flanking gene (within 1 kb) location in XYXZ genome.

**Additional file 19: Figure S4.** Length distribution of complete LTRs in XYXZ.

**Additional file 20: Figure S5.** Distribution of ultra-long genes in XYXZ genome.

**Additional file 21: Table S7.** Statistics for gene function annotation.

## Data Availability

Sequencing data generated for this project are achieved at National Genomics Data Center (NGDC) with BioProject number PRJCA012191, under accession code CRA008357.

## Abbreviations

XYXZ: Xiangyaxiangzhan
R498: Shuhui498
ZS97: Zhenshan97
MH63: Minghui63
TEs: Transposable elements
CRL: Continuous long reads
CCS: Circular consensus sequencing
SVs: Structural variations
LTRs: Long terminal repeat retrotransposons
ONT: Oxford nanopore technologies
Hi-C: High-throughput chromosome conformation capture
NR: Non-Redundant
InterPro: Protein domains and functional sites
KEGG: Kyoto encyclopedia of genes and genomes
tRNA: Transfer RNA
rRNA: Ribosomal RNA
snRNA: Small nuclear RNA
miRNA: MicroRNA
