# Supplementary figures and images for: Chromosomal-level genome assembly of the high-quality Xian/Indica rice (Oryza sativa L.) Xiangyaxiangzhan
Source: BMC Plant Biol. 2023 Feb 14;23:94. doi: 10.1186/s12870-023-04114-0 (PMC9926808; doi:10.1186/s12870-023-04114-0)

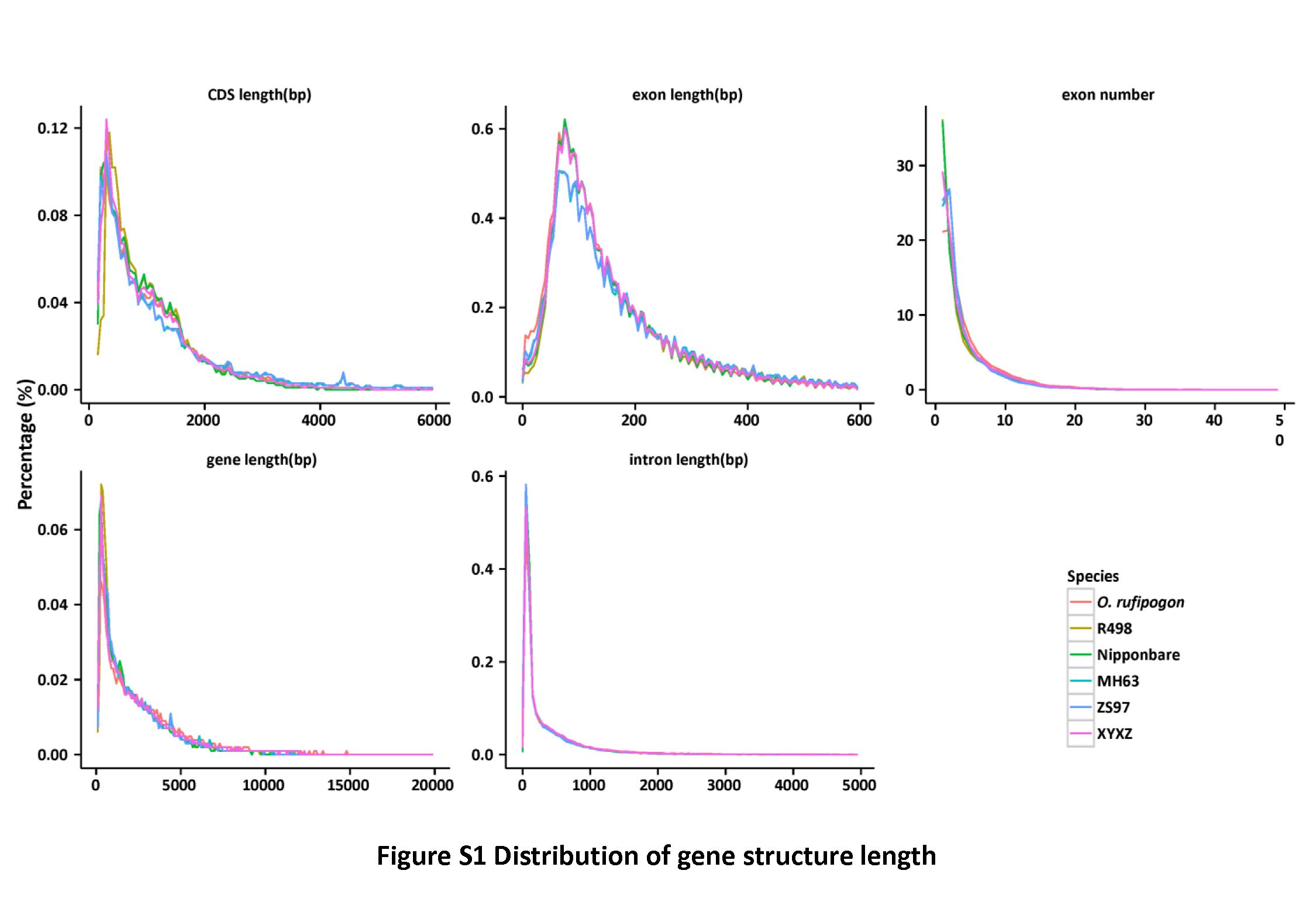

Supplement: Supplementary file 6 — Additional file 6: Figure S1. Distribution of gene structure length. [file 12870_2023_4114_MOESM6_ESM.tiff]

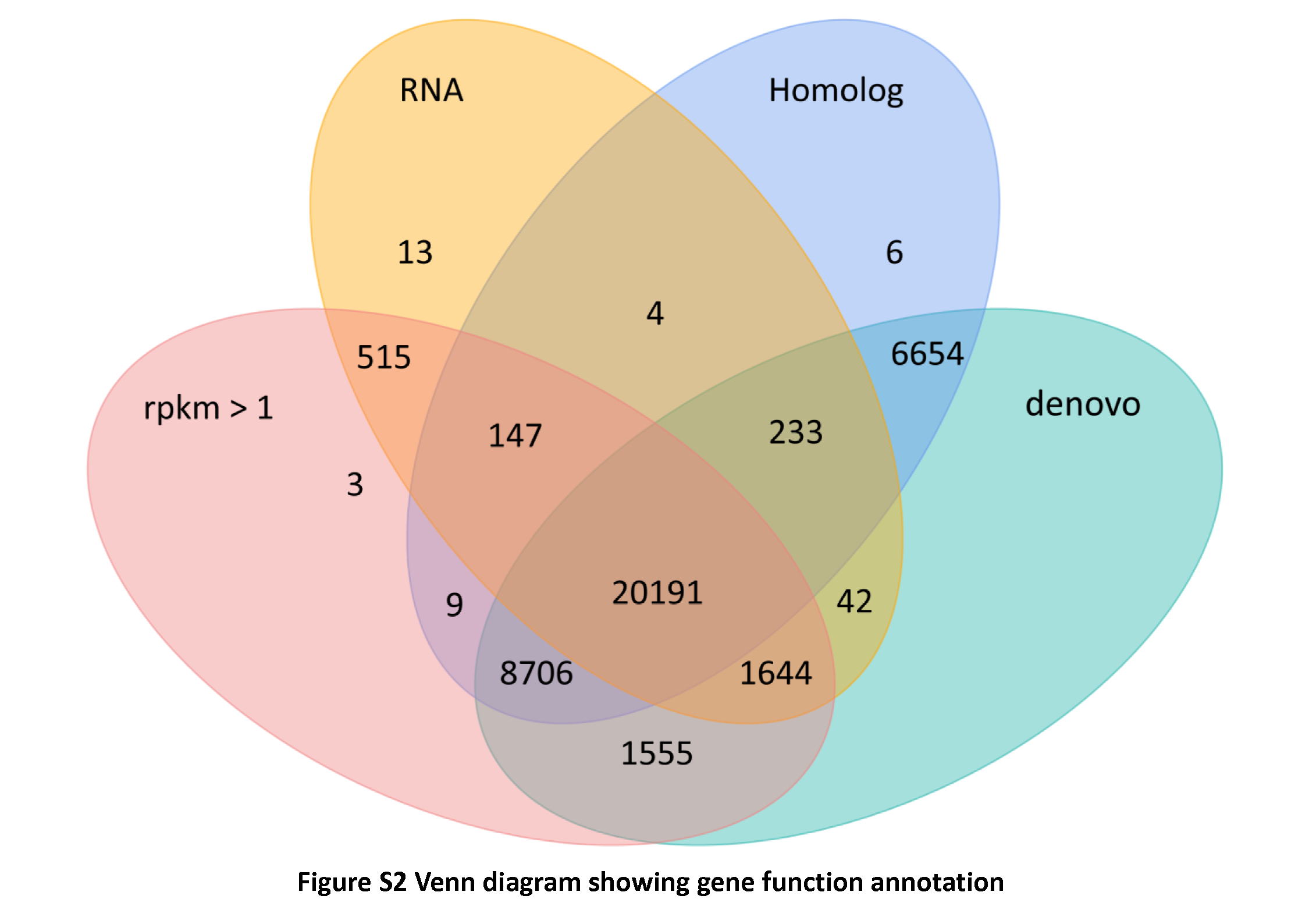

Supplement: Supplementary file 8 — Additional file 8: Figure S2. Venn diagram showing gene function annotation. [file 12870_2023_4114_MOESM8_ESM.tiff]

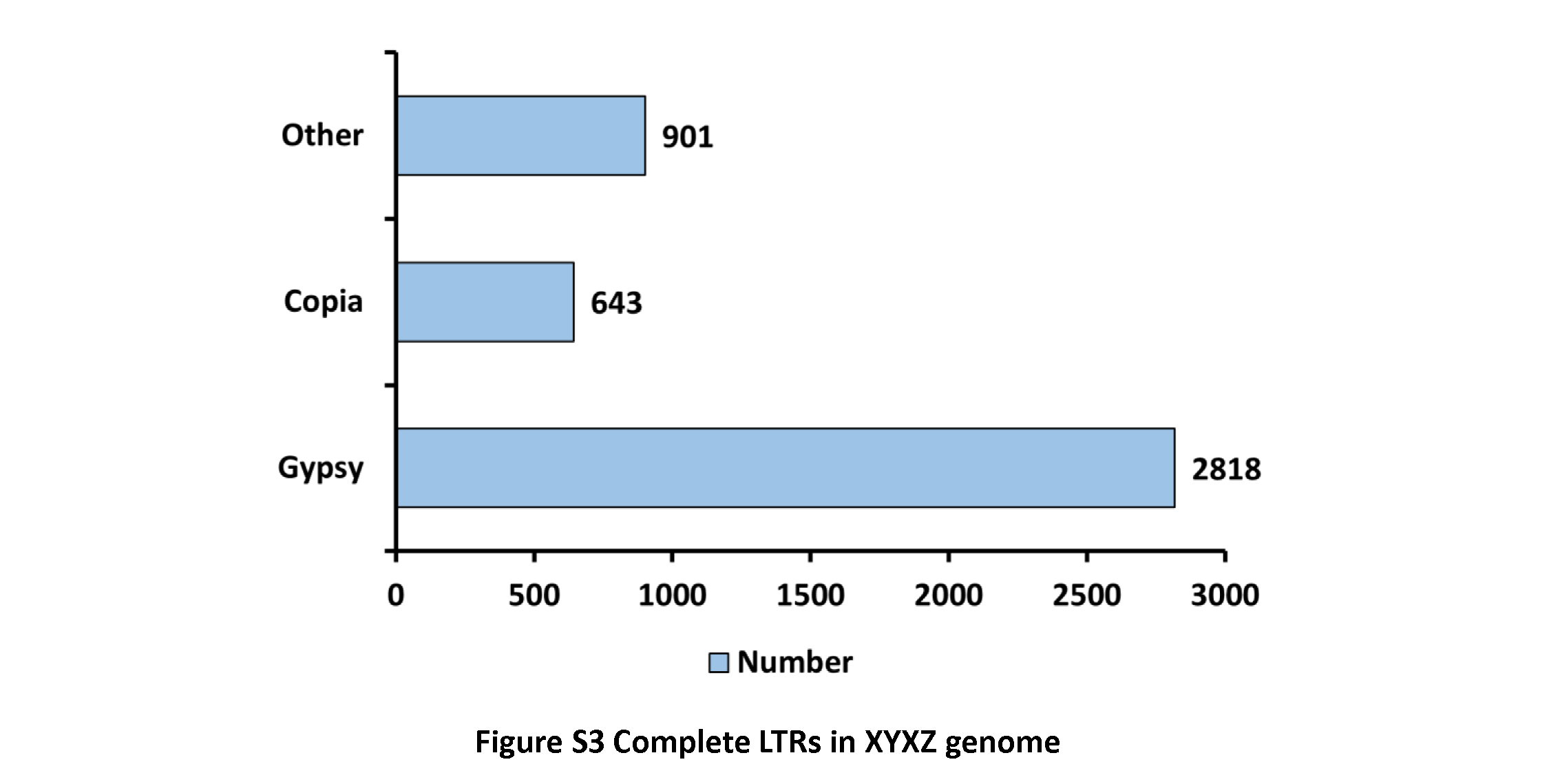

Supplement: Supplementary file 17 — Additional file 17: Figure S3. Complete LTRs in XYXZ genome. [file 12870_2023_4114_MOESM17_ESM.tiff]

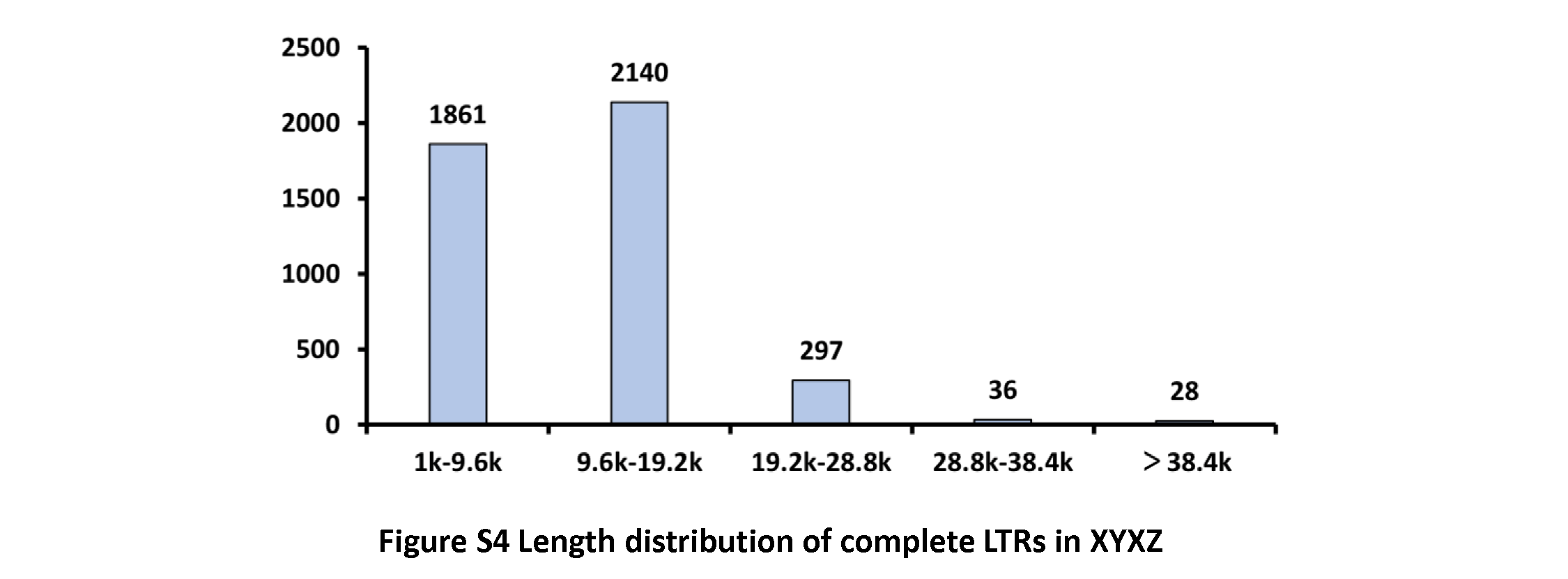

Supplement: Supplementary file 19 — Additional file 19: Figure S4. Length distribution of complete LTRs in XYXZ. [file 12870_2023_4114_MOESM19_ESM.tiff]

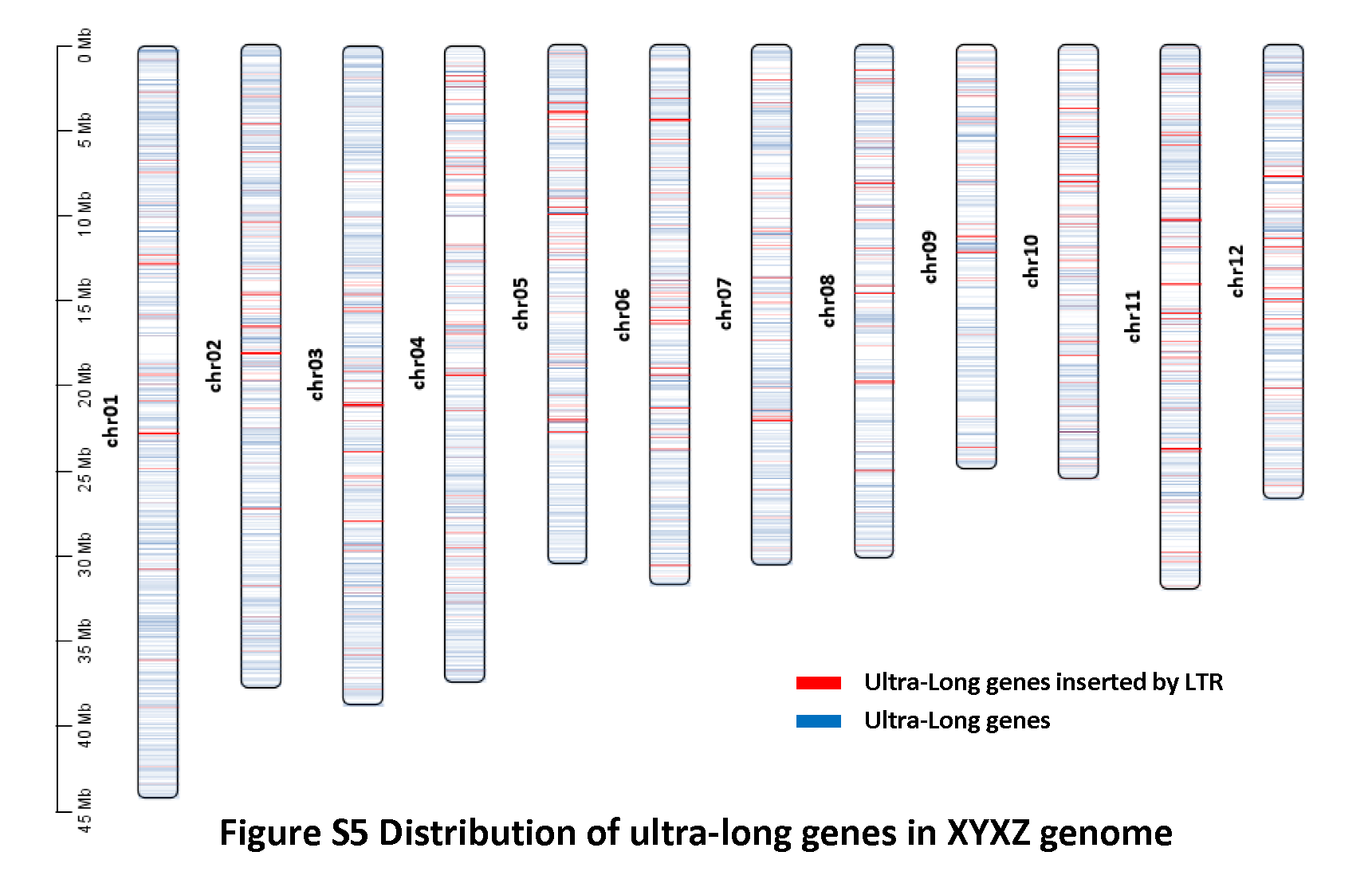

Supplement: Supplementary file 20 — Additional file 20: Figure S5. Distribution of ultra-long genes in XYXZ genome. [file 12870_2023_4114_MOESM20_ESM.tif]
